# A Case of Autosomal Dominant Osteopetrosis Type 2 with a *CLCN7* Gene Mutation

**DOI:** 10.4274/jcrpe.galenos.2019.2018.0229

**Published:** 2019-11-22

**Authors:** Sol Kang, Young Kyung Kang, Jun Ah Lee, Dong Ho Kim, Jung Sub Lim

**Affiliations:** 1 Korea Cancer Center Hospital, Clinic of Pediatrics, Seoul, Republic of Korea

**Keywords:** Osteopetrosis, bone density, osteoclast, sclerosis, mutation

## Abstract

Osteopetrosis is a rare genetic disease characterized by increased bone density and bone fractures due to defective osteoclast function. Autosomal dominant osteopetrosis type 2 (ADO-2), Albers-Schonberg disease, is characterized by the sclerosis of bones, predominantly involving the spine, pelvis and the base of the skull. Here, we report a typical case of osteopetrosis in a 17.7-year-old male who carries a heterozygous c.746C>T mutation in exon 9 in the *chloride voltage-gated channel 7 (CLCN7)* gene. The patient’s spine showed multiple sclerotic changes including sandwich vertebra. His father had the same mutation but his skeletal radiographs were normal. This is the first reported case of ADO-2, confirmed by genetic testing in a Korean patient.

**What is already known on this topic?**

Autosomal dominant osteopetrosis type 2 (ADO-2) is the benign form of osteopetrosis, which is characterized primarily by vertebral endplate thickening and includes increased cortical bone volume with fragile bones and multiple fractures later in life. In families with ADO-2, the penetrance ranges from 60 to 90%.

**What this study adds?**

This is the first case of autosomal dominant ADO-2 with a confirmed mutation in the *CLCN7* gene in Korea. The patients showed typical radiologic findings of ADO-2 with hearing loss. However, the father with the same mutation was asymptomatic, with no clinical or radiologic signs. Thus, we conclude that the exact prevalence is not known.

## Introduction

Osteopetrosis, also known as marble bone disease, is an extremely rare bone disease characterized by increased bone mineral density, where bones are prone to be fractured due to defective osteoclast function despite the increased bone mineral density (1,2,3). According to The Nosology Group of the International Skeletal Dysplasia Society, osteopetrosis is classified based on clinical features, mode of inheritance and molecular mechanism (2). There are various clinical features and genes involved in different types of osteopetrosis. Inheritance type can be autosomal recessive, autosomal dominant or X-linked. Autosomal recessive osteopetrosis (ARO), which is a malignant form of osteopetrosis that can be seen in infancy, can result in growth failure and increased frequency of fractures. Patients with ARO suffer from anemia and recurrent infections. It is thought that this is due to expansion of the bone which leads to narrowing of the bone marrow space and results in extramedullary hematopoises. Some patients with ARO also suffer from blindness, facial paralysis, and deafness, due to pressure on the cranial nerves by the narrowing of spaces due to bone expansion (1,2,3).

However, patients with autosomal dominant osteopetrosis (ADO), which is the benign form of osteopetrosis, may present with no symptoms of ADO and most of them are found incidentally. ADO type 2 (ADO-2), also known as Albers-Schonberg disease, is characterized primarily by vertebral endplate thickening “sandwich vertebrae” and includes increased cortical but normal cancellous bone volume, and fragile bones with multiple fractures later in life (4,5,6,7). The most common cause of ADO-2 is the presence of inactivating mutations in the *chloride channel 7* (*CLCN7*) gene, which results in ineffective, osteoclast-mediated bone resorption through disrupted acidification of the osteoclast resorption lacunae, that in turn prevents degradation of the mineral component of the bone (3). Here, we present a case of ADO-2 in an adolescent male, who carried a heterozygous gene mutation in *CLCN7*.

## Case Report

A 17.7-year-old male was referred to our hospital due to sclerotic changes in bony structures. Approximately one month prior to referral, the patient started complaining of pain in the right shin. X-rays in a local clinic revealed a generalized increase in bone density.

The patient’s history revealed that he weighed 3.8 kg (75^th^ percentile) at birth. He had no history of chronic diseases such as hypertension, diabetes or hepatitis. The patient and his family, including his parents and younger sister had no history of bone fractures. His aunt was suspected of having a bone-related disease, but she did not present for examination. The patient suffered from chronic otitis media and was diagnosed with partial hearing loss when he was 16 years old. On physical and neurological examination, no specific findings were noted. His current height and weight were 170.6 cm (50^th^ percentile) and 69.0 kg (75^th^ percentile), respectively.

**Figure 1:**
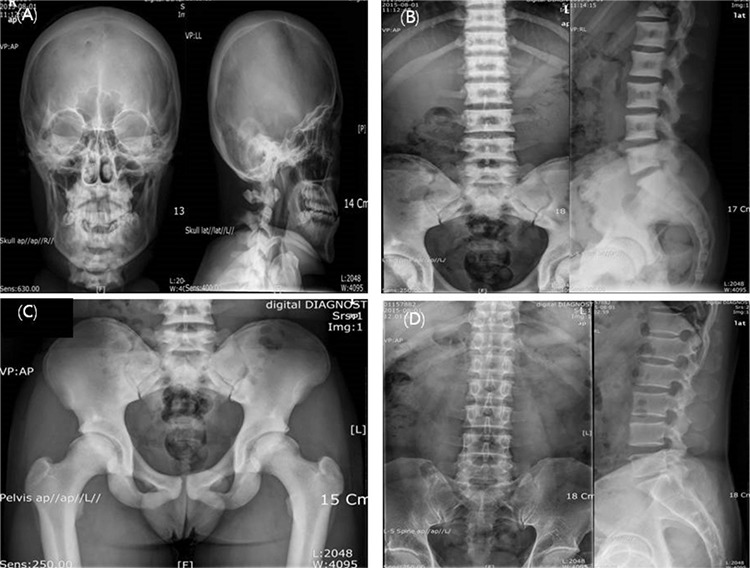
A) X-rays of the skull showing generalized increase in bone density. The sclerosis is more prominent in the base of the skull. B) Typical end-plate thickening and sclerosis producing the classic “sandwich vertebrae” appearance. C) Sclerosis in the iliac wings, acetabuli and femur heads. However, typical “bone-within-bone” appearance cannot be noted in the patient. D) The patient’s father showed normal bone density

Plain radiographs showed a generalized increase in bone density involving the skull, vertebrae and pelvis. X-rays of the skull showed thickening and increased skull-base density (Figure 1A). X-rays of the spine showed typical end-plate thickening and sclerosis producing the classic “sandwich vertebrae” appearance (Figure 1B). Sandwich vertebra is a radiologic finding in which the endplates are densely sclerotic, resulting in the sandwich appearance. X-rays of the pelvis showed the “bone-within-bone” appearance, primarily in the iliac wings (Figure 1C). The other family members, including his younger sister, mother and father, showed normal bone density. Figure 1D shows normal bone appearance in the patient’s father. Bone mineral densitometry (BMD) of the antero-posterior lumbar spine vertebrae, L1-L4, was measured as 2.466 g/cm^2^ (Z-score=10.7) by dual-energy X-ray absorptiometry on a Lunar Prodigy (Lunar, Madison, WI, USA). The BMD of the left femoral neck, trochanter and Ward’s triangle were measured as 1.966 g/cm^2 ^(Z-score=7.0) (8), 1.825 g/cm^2^, and 1.943 g/cm^2^, respectively. Blood chemistry showed the following: serum albumin 4.4 g/dL (reference range 3.5-5.2 g/dL), total calcium 9.5 mg/dL (8.6-10.2 mg/dL), elevated phosphorus 5.0 mg/dL (2.7-4.5 mg/dL), ionized calcium 4.81 mg/dL (4.48-4.92 mg/dL), alkaline phosphatase: 108 U/L (40-129 U/L), sodium 145 mmol/L, potassium at 4.4 mmol/L, chloride 105 mmol/L and bicarbonate 28.4 mmol/L. The intact parathyroid hormone level was slightly elevated, being 79.5 pg/mL (reference range: 14-72 pg/mL), 25-hydroxy-vitamin D_3_ level was 25.7 ng/mL (insufficiency range: 10-30 ng/mL) and thyroid stimulating hormone 5.38 uIU/mL (reference range: 0.27-4.20 uIU/mL).

**Figure 2:**
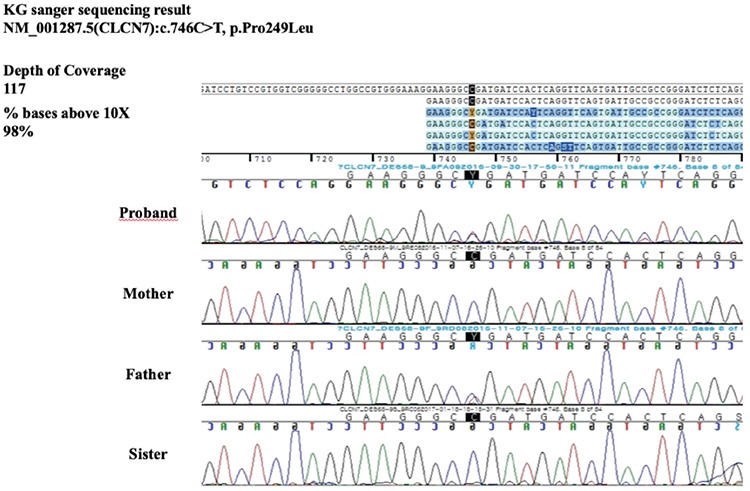
A heterozygous missense mutation was identified in the patient and his father. A heterozygous C to T transition is shown at position 746 in exon nine of *CLCN7* gene, changing a proline to leucine substitution at codon position 249

For evaluation of osteopetrosis, targeted gene panel sequencing was performed to check for the presence of pathogenic variants of multiple associated genes responsible for osteopetrosis. After informed consent, 3 mL of blood was obtained from the patient, sister and both parents. A library preparation was performed using the TruSight One Sequencing Panel (Illumina, Inc., San Diego, CA, USA), which enriches a 12-Mb region spanning 62,000 target exons of a total of 4,813 clinically relevant genes. Massively parallel sequencing was performed on the Illumina NextSeq platform. Sequence reads were mapped to UCSC hg19 standard base for comparative analysis. The results of targeted gene panel sequencing revealed heterozygous missense mutation c.746C>T (p.Pro249Leu) in exon 9 of the *CLCN7* gene in the proband, which was previously reported in a patient with ADO-2 (5): There was no pathogenic variant in other genes. Sanger sequencing confirmed the presence of this variant, and the same heterozygous variant was only found in the patient’s father (Figure 2). However, the father denied having any complaints including history of fracture, osteomyelitis, visual impairment and hearing problem. Radiographs of his bones were also normal (Figure 1D). We did not evaluate bone mineral density in the patient’s father, as his X-rays were of normal appearance.

## Discussion

This is the first case of autosomal dominant ADO-2 in Korea, with a confirmed mutation in the *CLCN7* gene. Previously, a case of infantile malignant *CLCN7*-related ARO, with neonatal thrombocytopenia was reported in Korea (9). There are two mutations in that case: (1) a deletion of an A at nucleotide 17631, in the paternally derived allele, causing a frame shift and a premature stop codon at codon 395; and (2) an intronic point mutation G23742A in the maternal allele.

ADO-2 is the most common form of osteopetrosis and is characterized by sclerosis, predominantly involving the spine, pelvis and base of the skull (4,5,6,7). The fragility of bones and dental abscess are common complications. The gene that is mutated in ADO-2 was reported to be localized on chromosome 16p13.3 and was later identified to be *CLCN7 *(5,6). The *CLCN7* gene encodes the chloride channel 7 protein subunit (ClC-7), which consists of 803 amino acids and plays a role in efficient proton pumping in the osteoclast ruffled membrane (3). Thus, patients with the *CLCN7* mutation have reduced bone resorption, which leads to osteopetrosis (10). Previously, over 70 different mutations in *CLCN7* have been identified in ADO-2 families and almost all cases have been associated with heterozygous mutations in the *CLCN7* gene (5,7,11,12).

The spectrum of *CLCN7*-related osteopetrosis includes infantile malignant ARO, intermediate autosomal osteopetrosis and ADO-2 (1). ADO-2 is a benign condition and the disease onset is usually in late childhood or adolescence. The diagnostic criterion for ADO-2 is osteosclerosis of the spine with a “sandwich vertebra” or “rugger-jersey” appearance. Most affected subjects have a “bone-within-bone” appearance, primarily in the iliac wings, but also in other long bones. Erlenmeyer-shaped femoral metaphyses, transverse bands of sclerosis and mild osteosclerosis in the base of the skull are often observed (1,2,3,4,7).

The complications of infantile malignant ARO include poor growth and fractures, with a life expectancy of fewer than 10 years. However, most ADO-2 cases are benign, with normal life expectancy. Long-term complications of ADO-2 include fractures in long bones or vertebrae, scoliosis, hip osteoarthritis and osteomyelitis (4). One study which included longitudinal data, suggested that the course of the ADO clinical phenotype worsens over time, especially with regard to fractures (13). Thus, vigorous physical activities should be avoided to prevent fractures and routine dental examination and oral hygiene are important to prevent osteomyelitis of the mandible. In treating fractures, orthopedic surgeons should pay special attention to delayed union or non-union fractures. Cranial nerve compression is a rare occurrence. Hearing loss and vision loss occur in fewer than 5% of affected subjects. In our case, the patient had hearing loss due to osteopetrosis, but we did not perform further evaluation in order to identify the cause of hearing loss in the right ear.

The prevalence of ADO-2 is estimated to be as low at 0.2-5.5 in 100,000 cases (14,15,16). However, asymptomatic carriers were reported with some mutations, and non-penetrance rates were 24 to 41%, depending on mutations, in families with ADO-2 and most of them are asymptomatic at younger ages (4,15,17). Furthermore, no *CLCN7* mutation could be found in up to 30% of patients presenting with a clinical phenotype of ADO (10,18). Given the reduced penetrance of the ADO phenotype, the spectrum of disease expression can range from radiographically unaffected gene carriers through skeletally affected yet asymptomatic subjects to severely affected patients with fractures which increase in severity over time, the prevalence of ADO-2 is likely to be higher (13). In our case, the father had the same mutation but was asymptomatic, with no clinical or radiologic signs. We believe his lack of symptoms was due to reduced penetrance of phenotype.

Therefore, it is important that *CLCN7* gene mutations be considered when patients have increased bone density, with radiologic findings such as “bone-within-bone” appearance. In future studies, we hope to perform genetic testing to confirm additional cases of asymptomatic Korean ADO-2 cases.
